# Combinatorial microenvironmental regulation of liver progenitor differentiation by Notch ligands, TGFβ, and extracellular matrix

**DOI:** 10.1038/srep23490

**Published:** 2016-03-30

**Authors:** Kerim B. Kaylan, Viktoriya Ermilova, Ravi Chandra Yada, Gregory H. Underhill

**Affiliations:** 1 Department of Bioengineering, University of Illinois at Urbana-Champaign, Urbana, IL, 61801, USA

## Abstract

The bipotential differentiation of liver progenitor cells underlies liver development and bile duct formation as well as liver regeneration and disease. TGFβ and Notch signaling are known to play important roles in the liver progenitor specification process and tissue morphogenesis. However, the complexity of these signaling pathways and their currently undefined interactions with other microenvironmental factors, including extracellular matrix (ECM), remain barriers to complete mechanistic understanding. Utilizing a series of strategies, including co-cultures and cellular microarrays, we identified distinct contributions of different Notch ligands and ECM proteins in the fate decisions of bipotential mouse embryonic liver (BMEL) progenitor cells. In particular, we demonstrated a cooperative influence of Jagged-1 and TGFβ1 on cholangiocytic differentiation. We established ECM-specific effects using cellular microarrays consisting of 32 distinct combinations of collagen I, collagen III, collagen IV, fibronectin, and laminin. In addition, we demonstrated that exogenous Jagged-1, Delta-like 1, and Delta-like 4 within the cellular microarray format was sufficient for enhancing cholangiocytic differentiation. Further, by combining Notch ligand microarrays with shRNA-based knockdown of Notch ligands, we systematically examined the effects of both cell-extrinsic and cell-intrinsic ligand. Our results highlight the importance of divergent Notch ligand function and combinatorial microenvironmental regulation in liver progenitor fate specification.

Microenvironmental regulation plays a key role in stem and progenitor cell fate/function in development. Within the liver, progenitor cell differentiation and bile duct morphogenesis are driven by spatially-dependent and temporally-sequenced cell–cell and cell–factor interactions coordinated by several signaling pathways, namely Notch and TGFβ^1^,^2^,^3^,^4^. During fetal liver development, a decreasing spatial gradient of TGFβ from the portal vein delineates cholangiocytic versus hepatocytic differentiation of bipotential liver progenitors^5^. NOTCH2 and JAG1 activity is required for both cholangiocytic fate specification and formation of mature intrahepatic bile ducts^6^,^7^,^8^,^9^,^10^. The importance of Notch in bile duct morphogenesis is further highlighted by Alagille syndrome, an autosomal dominant genetic disorder caused by mutations in *NOTCH2* or *JAG1* and associated with paucity of intrahepatic bile ducts, neonatal jaundice, cholestasis, and other abnormalities^11^,^12^,^13^,^14^. Moreover, extracellular matrix (ECM) proteins are known to regulate both fate specification and morphogenesis, as demonstrated by enhanced induction of bile duct epithelium by collagen I and Matrigel^15^,^16^, β1 integrin-mediated regulation of apicobasal polarity and subsequent morphogenesis by α1- and α5-containing laminin^17^, and activation of genes encoding ECM proteins by *Sox9*, a specific early marker of biliary epithelial cells, and *Sox4*^18^,^19^.

In addition to normal tissue development, duct morphogenesis also occurs in the adult liver in response to severe and chronic injury^20^. These so-called ductular reactions exhibit highly variable differentiation patterns and have been demonstrated to significantly contribute to proliferative response in the liver^21^. Notably, Notch signaling activation has been shown to be an important component of biliary regeneration in ductular reactions associated with chronic disease^22^. Changes in ECM have also been suggested to be involved as ECM remodeling occurs during ductular reactions in rodent models^23^,^24^. In particular, proliferating progenitor cells within these ductular reactions have been associated with the turnover of collagen I and the deposition of basement membrane structures containing laminin^25^. Collectively, despite many insights gained into the pathways involved in liver progenitor specification, the complete mechanistic details of the link between liver progenitor cell fate/function and liver duct morphogenesis as well as the combined impact of feedback between Notch, TGFβ, and ECM proteins remain unclear. Thus, an approach capable of simultaneously probing combinatorial microenvironmental regulation by cell–cell, cell–soluble factor, and cell–matrix interactions is required in order to define the functional overlap of these distinct pathways.

Mechanistic studies of Notch are difficult due not only to partially redundant function of receptors and ligands but also highly context-dependent pathway activity and function^26^,^27^,^28^. Cell–cell contact and binding of receptor (NOTCH1-4) to ligand (JAG1, JAG2, DLL1, DLL3, and DLL4) triggers cleavage of the receptor by ADAM metalloproteases and the γ-secretase complex, freeing the Notch intracellular domain to localize to the nucleus and mediate gene transcription through interactions with the DNA-binding protein RBPJ-k. Even in contexts for which key Notch ligand-receptor pairs have been identified, such as NOTCH2–JAG1 for Alagille syndrome, function remains highly dependent on cell type and microenvironmental context. In particular, the degree of Notch signaling activation can be substantially influenced by interactions with other pathways. For hepatocyte regeneration in the setting of chronic liver injury, for example, activation of the Wnt pathway in liver progenitor cells causes an inhibition of Notch signaling, preventing cholangiocyte differentiation and promoting hepatocyte differentiation^22^. Relevant *in vitro* methods for studying regulation by specific Notch receptors or ligands include ligand immobilization^29^,^30^, antibody-mediated functional blocking of specific receptors^31^,^32^, and treatment with soluble Notch ligand peptide^33^. Here, we exploited a cellular microarray platform, which exhibits well-defined material properties and unique capabilities for simultaneously examining multiple types of microenvironmental regulation^34^,^35^,^36^. Using this approach, we investigated liver progenitor differentiation within defined microenvironments consisting of systematically introduced soluble factors, ECM components, and cell–cell signaling ligands.

In this study, we demonstrate a cooperative role of Notch and TGFβ in liver progenitor fate specification, including unique effects of the Notch ligands *Jag1* and *Dll1* on the differentiation process. Utilizing a co-culture format together with shRNA-mediated knockdown of *Jag1* or *Dll1*, we explored the cell-extrinsic versus cell-intrinsic influence of these ligands. In addition, a cellular microarray platform was used to quantify microenvironmental regulation by five ECM proteins (collagen I, collagen III, collagen IV, fibronectin, and laminin) for all 32 (2^5^) possible combinations. We further adapted this microarray platform to include highly-functional Protein A/G-conjugated Notch ligands, showing induction of cholangiocytic differentiation by exogenous (cell-extrinsic) presentation of JAG1, DLL1, and DLL4 dependent on ECM and cell-intrinsic expression of *Jag1* and *Dll1*. In summary, our study of liver progenitor fate specification implicates combinatorial interactions between Notch, TGFβ and ECM proteins and further suggests that the Notch ligand *Dll1* may exhibit effects distinct from *Jag1* in hepatocytic and cholangiocytic differentiation.

## Results

### Cooperative effects of Notch and TGFβ on liver progenitor differentiation

**Figure 1:**
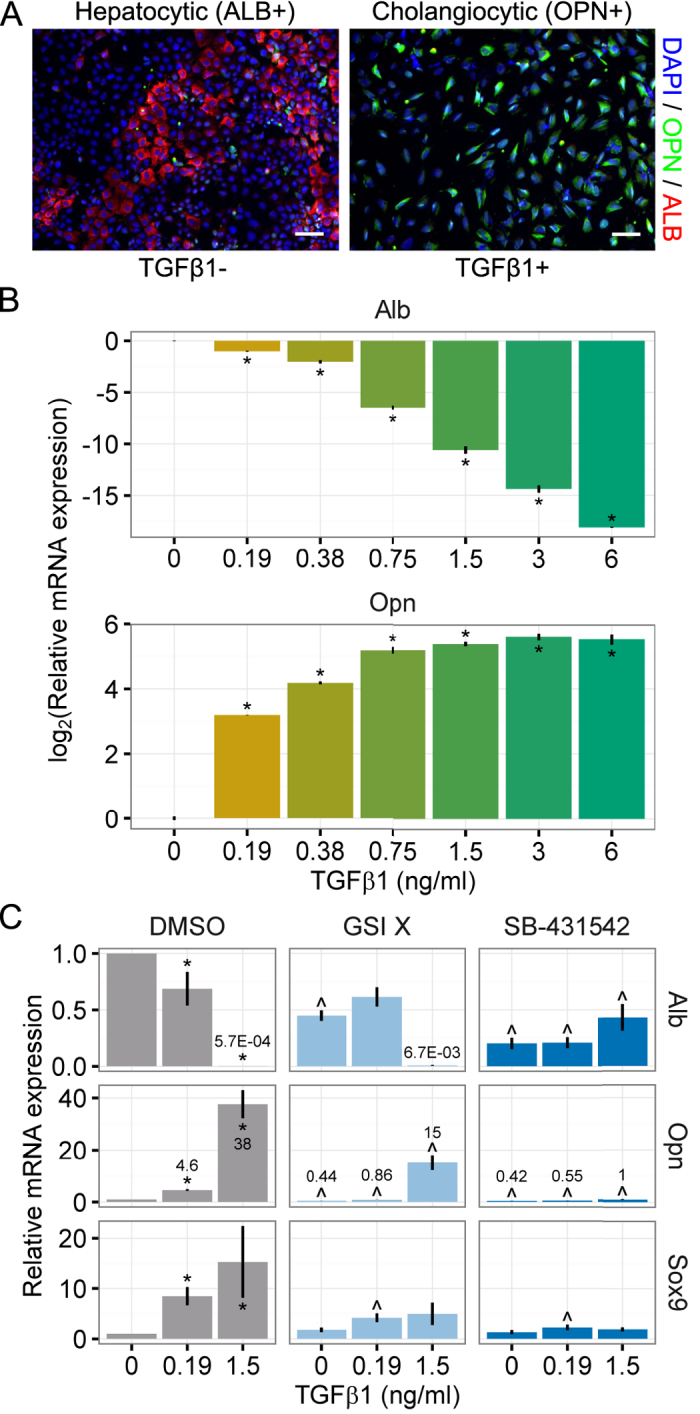
Liver progenitors differentiate into hepatocytes and cholangiocytes via TGFβ and Notch. (**A**) Micrographs of BMEL progenitor cells cultured under differentiation conditions (TGFβ1±). BMEL cells in TGFβ1+ were cholangiocytic (ALB−/OPN+) while those in TGFβ1− were hepatocytic (ALB+/OPN−). Scale bars are 50 μm. (**B**) qRT-PCR analysis of *Alb* and *Opn* mRNA transcripts in BMEL cells treated with increasing doses of TGFβ1. Student’s *t*-tests were performed against 0 ng/ml for each concentration of TGFβ1 with P-values indicated for P < 0.05 (*). (**C**) qRT-PCR analysis of *Alb, Opn*, and *Sox9* mRNA transcripts in BMEL cells treated with TGFβ1, γ-secretase inhibitor X (5 μM, GSI X), or SB-431542 (10 μM). For the DMSO treatment, Student’s *t*-tests were performed against 0 ng/ml for each concentration of TGFβ1 with P-values indicated for P < 0.05 (*). For the GSI X and SB-431542 treatments, Student’s *t*-tests were performed against equal TGFβ1 concentrations in the DMSO treatment with P-values indicated for P < 0.05 (^). Numeric callouts show *y*-axis values (not P-values). Data presented as mean ± s.e.m. with *n* = 3. log_2_ errors are relative. See also Supplemental Figs S1 and S2.

In order to systematically examine the microenvironmental regulatory mechanisms underlying liver progenitor differentiation, we used bipotential mouse embryonic liver (BMEL) 9A1 cells as a model liver progenitor cell type. These cells were derived from embryonic day 14 mouse embryos, can be induced to differentiate into hepatocytes or cholangiocytes *in vitro*, and have further been demonstrated to exhibit bipotential differentiation *in vivo*^37^,^38^. Previous efforts have utilized three-dimensional cell aggregate culture to induce hepatocytic differentiation of BMEL cells. To enable the series of studies implemented here, we first tested the capability of inducing BMEL cell differentiation within high-density two-dimensional monolayer culture. Under these differentiation conditions (i.e., high cell seeding density and reduced serum without insulin, IGF-2, and EGF), the BMEL cells committed to a hepatocytic fate, exhibiting an upregulation of albumin (ALB) protein (Fig. 1A). Consistent with the previously recognized role of TGFβ1 in cholangiocyte differentiation^2^,^5^, addition of TGFβ1 at the initiation of the differentiation cultures resulted instead in a commitment to a cholangiocytic fate, indicated by reduced expression of ALB and upregulation of osteopontin (OPN), a matricellular protein associated with ductular cholangiocytes but not hepatocytes^39^. We further examined the differentiation of BMEL cells in the presence of varied concentrations of TGFβ1. These experiments demonstrated a dose-dependent effect of TGFβ1 on inducing *Opn* mRNA expression and repressing *Alb* mRNA expression (Fig. 1B). In addition, TGFβ1 treatment increased mRNA expression of *Sox9*, a transcription factor known to be expressed during cholangiocyte differentiation *in vivo*, and at doses of 1.5 ng/ml and greater, TGFβ1 repressed the hepatocytic transcription factor *Hnf4a* (Supplemental Fig. S1). Taken together, these results are consistent with the role of TGFβ1 in driving cholangiocytic fate and suppressing hepatocytic fate. Next, we sought to determine if other pathways, in particular Notch signaling, act together with TGFβ1 to regulate the differentiation trajectory.

Treatment with an inhibitor of Notch signaling (γ-secretase inhibitor X, GSI X) partially suppressed cholangiocytic differentiation (*Opn* and *Sox9* mRNA expression) in a manner dependent on TGFβ1 dose (Fig. 1C). Specifically, at 1.5 ng/ml of TGFβ1, GSI X partially blocked *Opn* mRNA transcript expression (40.2 ± 7.74% of DMSO, P < 0.001), compared to a more substantial relative repression of *Opn* expression at 0.19 ng/ml (18.6 ± 0.74% of DMSO, P < 0.001). In comparison, treatment with SB-431542, an inhibitor of TGFβ signaling, resulted in the near complete inhibition of *Opn* upregulation in response to TGFβ1 (2.75 ± 0.535% of DMSO, P < 0.001). In addition, SB-431542 unexpectedly downregulated *Alb* mRNA transcript expression at low TGFβ1 concentrations, which could potentially result from either off-target effects of this inhibitor^40^ or from a currently unidentified effect on autocrine signaling pathways. We also observed upregulation of mRNA transcripts of the Notch-related transcription factors *Hes1* and *Hey2* as well as the cholangiocytic marker *Ggt1* by treatment with 5.0 ng/ml of TGFβ1; both GSI X and SB-431542 downregulated all three mRNA transcripts (Supplemental Fig. S2). Expression of the hepatocytic transcription factor *Cebpa* was reduced by TGFβ1 treatment; SB-431542 (but not GSI X) served to upregulate mRNA transcript expression to levels similar to the condition without TGFβ1 (Supplemental Fig. S2).

**Figure 2:**
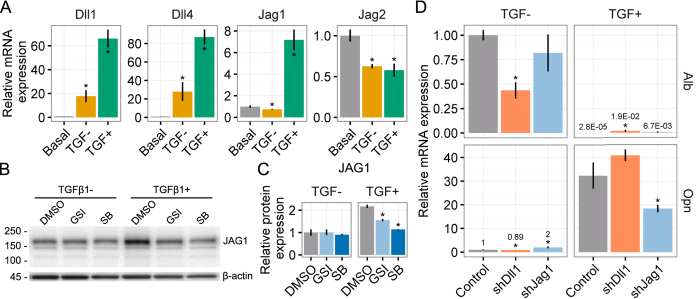
Jag1 and TGFβ1 coordinate cholangiocytic fate specification. (**A**) qRT-PCR analysis of *Dll1, Dll4, Jag1*, and *Jag2* mRNA transcripts in BMEL cells under basal (growth) and differentiation (TGFβ1±) conditions. Student’s *t*-tests were performed against basal for TGFβ1±. (**B**) Representative immunoblot against JAG1 in BMEL cells under differentiation conditions (TGFβ1±). Cells were further treated with an equivalent volume of vehicle (DMSO), GSI X (5 μM, GSI), or SB-431542 (10 μM, SB). Molecular weight markers shown in kDa (left) and β-actin control at 45 kDa (bottom). (**C**) Quantification of JAG1 immunoblots described in (**B**). Student’s *t*-tests were performed against DMSO for TGFβ1±. (**D**) qRT-PCR analysis of *Alb* and *Opn* mRNA transcripts in BMEL cells infected with lentiviral shRNA constructs against a non-target sequence (control), *Dll1* (shDll1), and *Jag1* (shJag1). For shDll1 and shJag1, Student’s *t*-tests were performed against the same treatment condition (TGFβ1±) in control cells. Numeric callouts show *y*-axis values (not P-values). Data presented as mean ± s.e.m. with *n* ≥ 3. P-values indicated for P < 0.05 (*). See also Supplemental Figs S2 and S3.

Based on the chemical inhibition data implying potential cooperation between TGFβ and Notch signaling, our next series of experiments explored the expression and the functional relevance of Notch ligands in BMEL cells. BMEL cells upregulated mRNA transcripts for the Notch ligands *Dll1, Dll4*, and *Jag1* under differentiation conditions (TGFβ1±), particularly in TGFβ1+ (Fig. 2A). Notably, *Dll1* and *Dll4* were upregulated under hepatocytic (TGFβ1−) and cholangiocytic (TGFβ1+) differentiation conditions, although the upregulation was more pronounced in the presence of TGFβ1. In contrast, *Jag1* was exclusively upregulated in cholangiocytic (TGFβ1+) conditions. *Jag2* was not induced by either differentiation conditions or TGFβ1. As *Jag1* has been implicated in previous studies of liver progenitor fate specification, we assayed regulation of its protein product JAG1 by TGFβ1 (Fig. 2B) and confirmed upregulation by densitometry (Fig. 2C). Furthermore, both GSI X and SB-431542 blocked upregulation of JAG1 by TGFβ1, confirming positive feedback by both Notch and TGFβ signaling. Expression of β-actin remained unchanged by treatment with GSI X and SB-431542 (Fig. 2B and Supplemental Fig. 2). To assess the functional role of Notch ligands in TGFβ1-mediated cholangiocytic fate specification, we employed lentiviral vectors containing shRNA sequences against a non-target sequence (control), *Dll1* (shDll1), and *Jag1* (shJag1), and confirmed knockdown at both mRNA transcript (*Dll1* and *Jag1)* and protein levels (JAG1) by qRT-PCR analysis and immunoblot, respectively (Supplemental Fig. S3). Following differentiation induction, we observed morphological differences in shJag1 and shDll1 cells that suggested an altered response to TGFβ1 (Supplemental Fig. S3). Thus, we evaluated the effects of *Jag1* and *Dll1* knockdown on *Alb* and *Opn* mRNA expression in response to TGFβ1 (Fig. 2D). Although *Alb* expression was not significantly affected by the knockdown of *Jag1, Dll1* knockdown appeared to have a distinct effect on *Alb* expression. In particular, *Dll1* knockdown decreased *Alb* expression in TGFβ1− and increased *Alb* expression in TGFβ1+ relative to control cells. The most substantial effect on *Opn* expression was measured following treatment with TGFβ1. Upon differentiation in these conditions, *Opn* expression was reduced in *Jag1* knockdown cells but remained unaffected by knockdown of *Dll1*, further confirming the role of *Jag1* in cholangiocytic fate specification.

### ‘Knockdown co-cultures’ demonstrate distinct roles for Jag1 and Dll1 ligands

**Figure 3:**
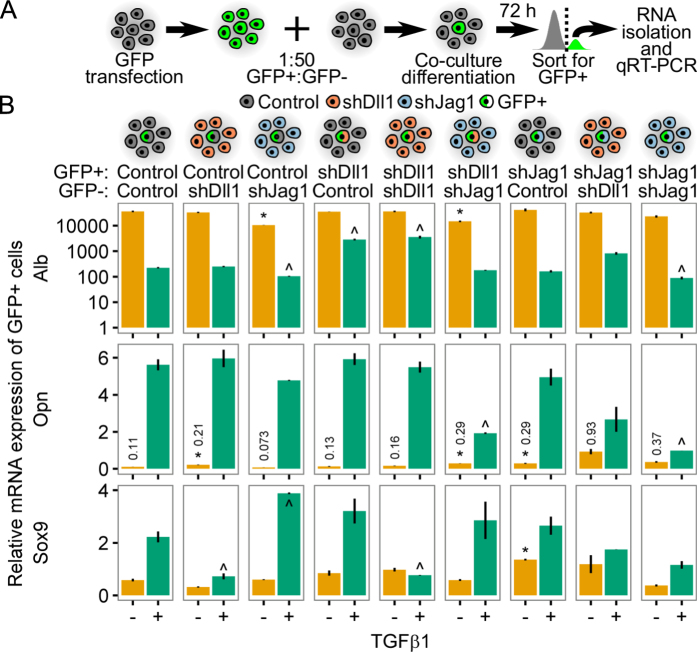
GFP+/GFP− co-cultures confirm distinct roles for Jag1 and Dll1. (**A**) Schematic of GFP+/GFP− co-culture experiment. GFP+ cells were generated using a GFP adenovirus and co-cultured at a 1:50 ratio with GFP− cells under differentiation conditions (TGFβ1±). GFP+ cells were collected after 72 h of culture by flow sorting for downstream RNA isolation and qRT-PCR analysis. (**B**) qRT-PCR analysis of *Alb, Opn*, and *Sox9* mRNA transcripts in GFP+ cells from co-cultures of every GFP+/GFP− combination of control-, shDll1-, or shJag1-infected BMEL cells. Results were normalized to expression in cultures grown under basal conditions in parallel with co-cultures. For each gene, Student’s *t*-tests were performed against Control^GFP+^ (Control^GFP−^) for every combination of GFP− and GFP+ cells. P < 0.05 indicated separately for TGFβ1− (*) and TGFβ1+(^). Numeric callouts show *y*-axis values (not P-values). Data presented as mean ± s.e.m. with n ≥ 3. See also Supplemental Fig. S4.

Experiments assessing bulk mRNA transcript and protein levels in response to chemical inhibition or genetic manipulation are unable to clarify whether Notch ligands work by cell-intrinsic (cell-autonomous) or cell-extrinsic (non-cell-autonomous) mechanisms. We designed a GFP+/GFP− co-culture platform to address these gaps in knowledge and methodology (Fig. 3A). GFP+ BMEL cells were generated by adenoviral transduction and mixed at a 1:50 ratio with GFP− cells. This ratio was selected to balance the need to collect sufficient numbers of cells for endpoint analysis with the requirement that GFP+ cells not be in contact with one another during the differentiation protocol, a cell–cell interaction that would dilute the results of the assay. After 72 h under differentiation conditions (±1.5 ng/ml TGFβ1), GFP+ cells were spatially separated and primarily in contact only with GFP− cells (Supplemental Fig. S4). Approximately 100,000 GFP+ cells were then collected from co-cultures by flow sorting, from which 150–300 ng of RNA was isolated for downstream qRT-PCR analysis. Introduction of control-, shDll1-, or shJag1-infected BMEL cells into this co-culture platform allowed for the assessment of the impact of both cell-intrinsic (i.e., GFP+) and cell-extrinsic (i.e., GFP−) knockdown of Notch ligand.

qRT-PCR analysis of *Alb, Opn*, and *Sox9* mRNA transcripts in RNA isolated from GFP+ cells showed distinct roles for *Jag1* and *Dll1* (Fig. 3B). In agreement with the results from bulk cell cultures, shJag1^GFP+^ (shJag1^GFP−^) resulted in downregulation of *Opn* while shDll1^GFP+^ (shDll1^GFP−^) had minimal impact. In addition, shDll1^GFP+^ (shJag1^GFP−^) resulted in downregulation of *Opn* to a level similar to shJag1^GFP+^ (shJag1^GFP−^). These results imply that *Opn* upregulation results from the combined effect of cell-intrinsic and cell-extrinsic *Jag1* expression, and that *Dll1* may cooperate with *Jag1* to mediate this upregulation. Interestingly, shDll1^GFP+^ (Control^GFP−^) and shDll1^GFP+^ (shDll1^GFP−^) conditions independently exhibited elevated *Alb,* which is consistent with the relative increase in *Alb* expression in TGFβ1− for *Dll1* knockdown cells (Fig. 2D). These data suggest that *Dll1* may act in a cell-intrinsic manner to repress *Alb* expression in response to TGFβ1-induced differentiation. The relative expression levels of *Sox9* were generally consistent with *Opn*, with a few exceptions. In particular, *Sox9* was not upregulated in the Control^GFP+^ (shDll1^GFP−^) and shDll1^GFP+^ (shDll1^GFP−^) conditions following TGFβ1 treatment, suggesting that *Sox9* upregulation is most significantly dependent on cell-extrinsic *Dll1* signaling. Divergence from expected expression profiles was further visualized through control-normalized *Alb*/*Opn* and *Opn*/*Sox9* ratios (Supplemental Fig. S4).

### Cellular microarrays establish influence of ECM on progenitor fate

**Figure 4:**
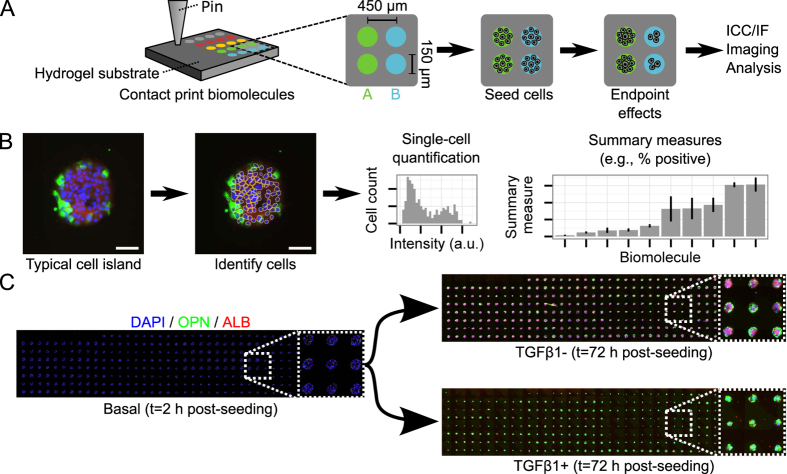
Cellular microarrays enable studies of combinatorial microenvironmental regulation. (**A**) Schematic of a cellular microarray experiment. Biomolecules and ECM proteins are patterned on a polyacrylamide hydrogel substrate using contact printing. Cells seeded on arrays adhere only to the patterned regions and are exposed to the deposited biomolecules and any experiment-specific soluble factors, fixed at endpoint, immunolabeled, imaged, and analyzed. (**B**) Analytical pipeline for cellular microarrays. Individual cells on islands are automatically identified by nuclear stain (DAPI) and associated with intensities in other channels, resulting in both single-cell and summary quantifications (e.g., percentage of cells positive for a marker) of results by deposited biomolecule and soluble factor treatment. Scale bars are 100 μm. (**C**) Experimental pipeline for cellular microarrays. BMEL cells are seeded for 2 h on arrays (sufficient to populate each patterned region), cultured under differentiation conditions (TGFβ1±) for 72 h, fixed, and labeled for nuclei, ALB, and OPN. Arrays shown are 18 × 4.5 mm (40 × 8 spots).

Both Notch and TGFβ signaling can be influenced by other microenvironmental signals, including the composition of the ECM. For example, ECM proteins can bind and sequester TGFβ, potentially contributing to the known gradient of TGFβ *in situ*^5^,^41^. In addition, integrin receptor crosstalk with both TGFβ and Notch signaling has been demonstrated in numerous cell contexts^42^,^43^,^44^. Thus, in order to further deconstruct the effects of these distinct microenvironmental signals, we utilized a cellular microarray approach (Fig. 4A). This platform enables a complete suite of capabilities to simultaneously assess the functional impact of both microenvironmental regulation (cell–cell, cell–ECM, cell–soluble factor) and genetic factors via shRNA knockdown. Further, here we have developed an analytical pipeline that facilitates both single-cell and summary quantifications through automated image analysis (Fig. 4B). Using identical differentiation protocols, BMEL cell fates on cellular microarrays were consistently similar to previous bulk observations (Fig. 4C).

**Figure 5:**
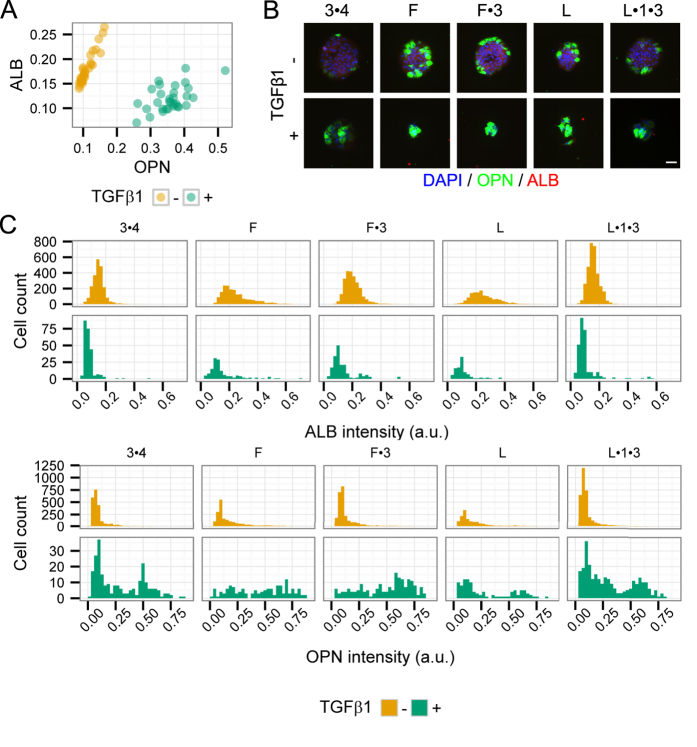
Microenvironmental regulation of liver progenitor differentiation by ECM proteins. (**A**) Scatter plot of ALB intensity against OPN intensity by TGFβ1 treatment. Each point represents a single arrayed ECM protein combination. (**B**) Immunofluorescence micrographs of selected ECM conditions. Scale bar is 50 μm. (**C**) Single-cell histograms of ALB and OPN label intensity for selected ECM proteins by TGFβ1 treatment. Data presented as mean ± s.e.m. with n = 3. Abbreviations: 1 = collagen I, 3 = collagen III, 4 = collagen IV, F = fibronectin, L = laminin. Combinations denoted by “•”, e.g., “1•3•4” denotes an ECM combination containing collagen I, III, and IV. See also Supplemental Figs S5, S6 and S7.

To specifically examine cell–ECM interactions, we adapted a previously-published array design and fabricated cellular microarrays incorporating all 2^5^ combinations of collagen I, collagen III, collagen IV, fibronectin, and laminin^34^. These ECM proteins were selected for their variable expression and function during hepatogenesis in the fetal and neonatal liver^45^. Following differentiation induction in the array format, staining and quantification of cell nuclei and differentiation markers was performed to evaluate cell number and degree of differentiation per ECM condition. Consistent with cell density observations in bulk cultures (Supplemental Fig. S3), treatment with TGFβ1 led to a relative decrease in cell numbers compared to untreated differentiation conditions (Supplemental Fig. S5). Quantification of ALB and OPN immunolabel intensity versus cell number demonstrated relative increases in marker intensity not correlated with cell number (Supplemental Fig. S5). In agreement with bulk experiments, ALB and OPN label intensity showed stratification by TGFβ1 treatment while the variation within each soluble treatment condition reflected the impact of ECM composition (Fig. 5A). We further quantified the percentage of cells positive for ALB and OPN following either untreated (TGFβ1−) or treated (TGFβ1+) differentiation conditions. These data demonstrate a large variation of ALB+ cell percentage by ECM and regardless of TGFβ1 treatment, indicating that ECM composition can play a role in regulating ALB expression (Supplemental Fig. S6). However, the profile of OPN+ cell percentage was more substantially influenced by TGFβ1 treatment with ECM composition having a less pronounced effect on OPN expression within the soluble treatment conditions (Supplemental Fig. S6). Regarding specific ECM components, fibronectin and laminin (and arrayed conditions containing either) were highly represented in the conditions with the highest percentage of ALB+ cells while collagen IV was predominant in conditions with lower percentages of ALB+ cells. These observations were further confirmed by main and interaction effects from full factorial multiple regression analysis (Supplemental Fig. S7). From this large-scale dataset, we have selected five arrayed conditions to illustrate the single-cell quantification capabilities and distinct profiles observed. Micrographs of these arrayed conditions not only confirm summary measure conclusions but also exhibit distinct cellular populations stratified in particular by OPN (Fig. 5B). Single-cell quantification highlights the observed variance in both cell count (histogram height) and ALB or OPN label intensity (Fig. 5C). In particular, we observed normal-like (ALB for 3•4 in TGFβ1−), Poissonian (ALB for F in TGFβ1−), uniform (OPN for F in TGFβ1+), and bimodal distributions (OPN for L•1•3 in TGFβ1+).

### Notch ligand microarrays demonstrate cell-extrinsic and cell-intrinsic effects on progenitor fate

**Figure 6:**
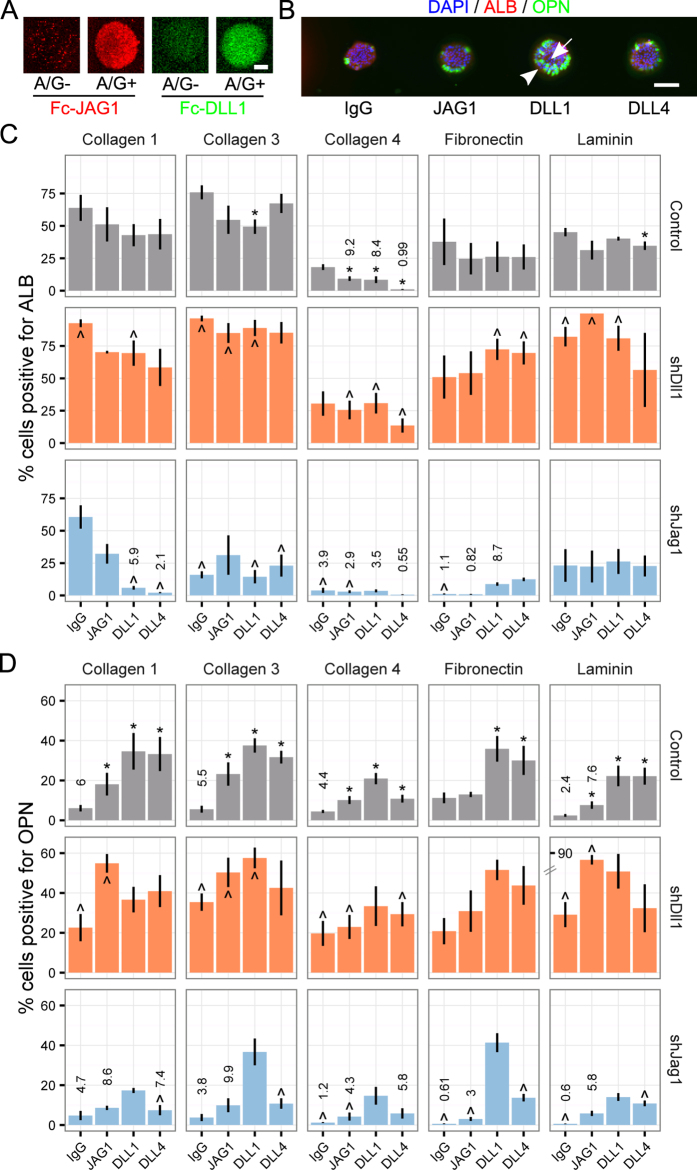
Arrayed Notch ligands drive cholangiocytic fate specification. (**A**) Immunolabeling of arrayed Fc-recombinant JAG1 and DLL1. Scale bar is 50 μm. (**B**) Immunofluorescence micrograph showing BMEL cells in TGFβ1−. Arrowhead shows spatial specificity of cholangiocytic (OPN+) differentiation at the edge of the island surrounding an OPN—core. Scale bar is 150 μm. (**C**) ALB quantification of shRNA-infected BMEL cells in TGFβ1− on five ECM proteins. (**D**) OPN quantification of shRNA-infected BMEL cells in TGFβ1− on five ECM proteins. Break in *y*-axis applies only to bar for JAG1/laminin/shDll1 condition. Data presented as mean ± s.e.m. with n ≥ 3. Hypothesis testing in (**C,D**) was performed as follows: For control cells, Student’s *t*-tests were performed against IgG for each arrayed Notch ligand within each ECM condition with P-values indicated for P < 0.05 (*). For shDll1 and shJag1 cells, Student’s *t-*tests were performed against the corresponding arrayed Notch ligand for control cells, again within each ECM condition with P-values indicated for P < 0.05 (^). See also Supplemental Figs S8 and S9.

In order to systematically investigate the effect of distinct Notch ligands on liver progenitor fate specification, we adapted the microarray platform to present Notch ligands. Specifically, we designed an array containing Fc-recombinant JAG1, DLL1, and DLL4. Notch ligands are known to require clustering to function both *in situ* or when adsorbed to or deposited on a substrate^30^,^46^. We used Fc-recombinant Notch ligands pre-conjugated to Protein A/G at a molar ratio of 1:6 as a means of mediating clustering and retention in the hydrogel substrate and improving cellular recognition. Immunolabeling of arrayed JAG1 and DLL1 showed increased signal and a less diffuse pattern when conjugated with Protein A/G (Fig. 6A). Arrayed Fc-recombinant, Protein A/G-conjugated JAG1, DLL1, and DLL4 was functional, stimulating BMEL cells towards cholangiocytic fates even in TGFβ1− conditions (Fig. 6B). We subsequently expanded the array design to include all five ECM proteins from the previous array experiments and also shRNA-infected cells. Using this array design, we quantified the percentage of cells positive for ALB (Fig. 6C) and OPN (Fig. 6D) in the absence of exogenous TGFβ1. Further, we additionally evaluated the effects of Notch ligands on ALB and OPN following TGFβ1 treatment (Supplemental Fig. S8). Collectively, these data further confirm the presence of ECM-specific effects; for example, collagen IV was less conducive to fate specification in agreement with the ECM-only experiments. In addition, extrinsic presentation of JAG1 resulted in a relative upregulation of OPN only in select conditions (namely collagens I, III, and IV) while DLL1 and DLL4 consistently triggered upregulation of OPN.

**Figure 7:**
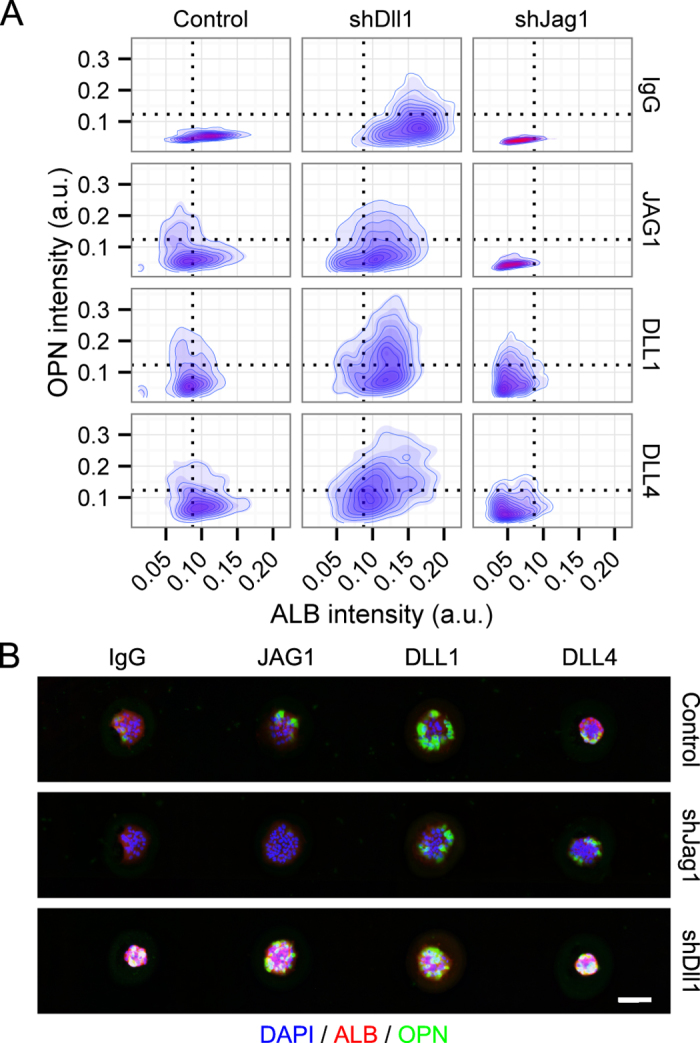
Imaging cytometry of Notch ligand arrays. (**A**) Contour maps showing imaging cytometry of shRNA-infected BMEL cells responding to Notch ligands on collagen III. Dotted lines show cutoffs determining cell positivity for both ALB (*x*-axis) and OPN (*y*-axis). (**B**) Immunofluorescence micrographs showing varying response to Notch ligand by shRNA-infected BMEL cells. Scale bar is 150 μm.

We further explored potential combinatorial effects of cell-intrinsic ligand expression by using the Notch ligand arrays in combination with the shJag1 and shDll1 BMEL cells previously evaluated in the bulk and co-culture experiments. These data show that shJag1 cells exhibited lower ALB and OPN expression (partially dependent on ECM context) as well as a decrease in the effect of arrayed Notch ligands, suggesting that the exogenous ligands cannot effectively compensate for the reduction in cell-intrinsic *Jag1* expression (Fig. 6C,D). In contrast, shDll1 cells demonstrated a different effect, in which both ALB and OPN increased compared to control cells (Fig. 6C,D). These conclusions were corroborated by the main effects from multiple regression analysis (Supplemental Fig. S9). In order to explore the potential presence of double-positive (ALB+/OPN+) cells, we further utilized the single-cell quantification data produced from this set of arrays to plot OPN label intensity versus ALB label intensity (Fig. 7A). These contour-density plots illustrate the combined effects of arrayed ligands and cell-based ligand knockdown. Most notably, these data demonstrate the presence of ALB+/OPN+ cells, which were primarily present following *Dll1* knockdown with an increased frequency in combination with exogenously-presented Notch ligand. This imaging cytometry-based quantification was correlated with cell morphologies observed in immunofluorescence micrographs (Fig. 7B).

## Discussion

**Figure 8:**
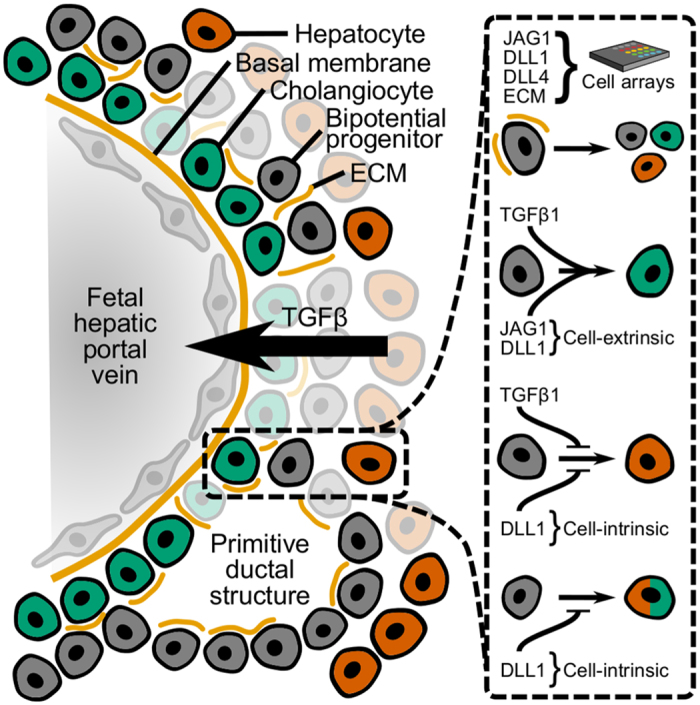
Schematic summary of approach and findings. Cellular microarrays enable controlled studies of the combined effects of microenvironmental signals, including TGFβ, Notch, and ECM. Analysis of BMEL cell differentiation within cellular microarrays and complementary co-culture formats is further suggestive of the following roles for distinct Notch ligands: TGFβ1 and *cell-extrinsic* Notch ligands (JAG1 and DLL1) cooperate to induce cholangiocytic fate; *cell-intrinsic* DLL1 plays a role in the suppression of hepatocytic fate in response to TGFβ1; and *cell-intrinsic* DLL1 inhibits the generation of double-positive (ALB+/OPN+) cells during differentiation.

In order to examine the complex regulatory mechanisms governing stem and progenitor fate specification, methods that enable the systematic perturbation of microenvironmental signals are required. In these studies, we have developed and applied a cohort of strategies to investigate the combined roles of TGFβ, Notch, and ECM in liver progenitor bipotential differentiation. A schematic representation of our overall approach and findings is illustrated in Fig. 8. Taken together, our results confirm that liver progenitor differentiation is influenced by both *Jag1* and TGFβ1 and we further illustrate numerous combinatorial effects of the Notch and TGFβ signaling pathways. In particular, using a GFP+/GFP− co-culture approach, we separated the cell-intrinsic and cell-extrinsic functions of Notch ligands and showed distinct roles for *Jag1* and *Dll1* with shRNA knockdown. Additionally, we established ECM-specific effects using a cellular microarray platform that further formed the basis for the fabrication of Notch ligand microarrays. Exogenous presentation of Protein A/G-conjugated Fc-recombinant Notch ligands (JAG1, DLL1, and DLL4) in this microarray platform induced cholangiocytic differentiation and further produced ALB+/OPN+ double-positive cells when combined with *Dll1* knockdown.

Both Notch and TGFβ have been demonstrated to be involved in the differentiation of cholangiocytes and the formation of bile ducts^1^,^2^. Transcriptional profiling of HBC-3 murine liver progenitors has previously revealed upregulation of family members from both of these pathways during cholangiocytic specification^47^. Here, we demonstrate not only direct signaling effects but also inter-pathway feedback, as evidenced by the higher TGFβ1 threshold for cholangiocytic specification with GSI X treatment (Fig. 1C), the upregulation of *Dll1, Dll4*, and *Jag1* mRNA transcripts by TGFβ1 (Fig. 2A), and downregulation of JAG1 by inhibitors of both Notch and TGFβ (Fig. 2C). These observations are consistent with studies of other tissues in which *Jag1* was upregulated by TGFβ through protein-protein interactions between SMAD3 and the Notch intracellular domain^48^,^49^. Moreover, this suggests the known periportal gradient of TGFβ may also serve to upregulate JAG1 and other Notch pathway members during ductal plate patterning. Data regarding *Jag1, Dll1*, and other Notch ligands must be synthesized with the known behaviors and functions of Notch receptors. In particular, Ortica *et al*. recently showed that *Notch2* and *Notch4* maintain progenitor state in BMEL cells whereas *Notch3* was associated with a hepatocytic morphology^50^. Cell type-specific (conditional) inducible mouse models show that *Notch2* (but not *Notch1*) is indispensable for cholangiocytic differentiation and furthermore coordinates patterning of the ductular network^7^,^51^,^52^.

Notch pathway activity in the liver is highly sensitive to dosing and spatial localization of both receptor and ligand^3^,^6^,^10^. This interplay is further underscored by the observation that *Jag1*^*−/*^+/*Notch2*^*−/*^+ double heterozygous mice exhibit features of Alagille syndrome^8^. In canonical Notch signaling, Notch ligands act as binding partners for the Notch receptors, a cell-extrinsic mechanism through which transcriptional activity occurs in the receiving (and not ligand-presenting) cell. Our co-culture data imply that *Jag1* and *Dll1* may work together through a combination of cell-extrinsic and cell-intrinsic means in cholangiocyte specification. For instance, both the shDll1^GFP+^ (shJag1^GFP−^) and shJag1^GFP+^ (shJag1^GFP−^) conditions exhibited similar reductions in OPN expression compared to conditions exhibiting only extrinsic knockdown: Control^GFP+^ (shJag1^GFP−^) and Control^GFP+^ (shDll1^GFP−^). Consistent with this trend, the shJag1^GFP+^ (shDll1^GFP−^) condition exhibited a moderate reduction of OPN, though not statistically significant (P = 0.118). Taken together, these data are suggestive of potential overlaps in function and mechanism for *Jag1* and *Dll1*, specifically for cholangiocyte differentiation.

Notably, both exogenous (cell-extrinsic) presentation of DLL1 as well as *Dll1* knockdown elicited increases in cholangiocytic specification in the Notch ligand arrays, in the absence of exogenous TGFβ (Fig. 6). It is possible that the cell-intrinsic effects observed are due to ligand intracellular domain signaling. Specifically, the Notch ligand intracellular domain is cleaved in the same manner as Notch receptors^53^,^54^ and is furthermore capable of nuclear translocation. The intracellular domain of DLL1 in particular is known to modulate SMAD-dependent transcription^55^,^56^. Further, these results are suggestive of the possibility that DLL1 expression may influence either Notch receptor expression or Notch signaling activity, effects that are observed in numerous contexts of lateral inhibition^27^,^57^,^58^ but have not previously been reported for liver differentiation and bile duct formation. The additional functionality of DLL4 may indicate some further redundancy with DLL1 which would require simultaneous knockdown of both ligands for further investigation.

In our ECM arrays, fibronectin and laminin were the most conducive to fate specification, particularly in TGFβ1−, while collagen IV was less conducive (Supplemental Figures S6 and S7), providing evidence that liver progenitor differentiation integrates ECM cues. Laminin and collagen IV are both main components of the basement membrane while fibronectin is largely expressed in the mesenchyme surrounding the portal vein^45^. Our observations are in general agreement with past studies: Tanimizu *et al*. showed induction of biliary cyst formation by laminin but not collagen IV while Yanai *et al*. observed ~220-fold induction of *Ck19* by the combination of collagen I and fibronectin (cf., <20-fold for collagen I alone)^15^,^16^. Tanimizu *et al*. further demonstrated that α1-containing laminin is sufficient for cholangiocytic fate specification while α5-containing laminin is necessary for bile duct formation^17^. As our formulation of laminin contained multiple subchains, future studies could delineate functional roles for each subchain in both co-cultures and arrays. In addition, based on the variations in cell number (and the corresponding size of the cell islands on the array) that we observed on distinct ECM conditions, future efforts could aim to exploit the microarray platform to directly examine potential synergistic or antagonistic interactions between ECM composition and cell–cell contacts during differentiation. Furthermore, one limitation of the cell microarray platform is the difficulty in evaluating numerous phenotypic markers simultaneously. Our array results presented here focused on the expression of ALB and OPN as characteristic markers of hepatocytic and cholangiocytic fates, respectively. Future efforts could build on these results by scaling-up relevant conditions and performing broader analyses of phenotypic marker expression and signaling pathway activation within distinct microenvironments.

Recent studies of liver progenitor differentiation and bile duct morphogenesis have revealed important details regarding the spatiotemporal dynamics of Notch signaling in the liver. Zong *et al*. demonstrated that Notch plays a role in differentiation, in addition to morphogenesis, and further found that Notch activity precedes differentiation of the first layer of the ductal plate^1^. Additionally, Hofmann *et al*. showed JAG1 in the portal mesenchyme controls ductal plate patterning but not fate specification^6^. Our data is consistent with a model of differentiation and early ductal plate formation that integrates feedback from multiple Notch ligands (namely *Jag1* and *Dll1*) expressed on progenitor cells. It is possible that both JAG1 in the mesenchyme and TGFβ induce JAG1 in progenitors as part of the specification process, consistent with lateral induction^59^,^60^.

In summary, our study highlights the importance of context-dependent Notch and TGFβ signaling as well as the integration of microenvironmental cues (namely ECM proteins) in liver progenitor differentiation. The effect of specific receptor-ligand interactions remains uncertain but could be investigated through presentation of Notch receptors in arrays or genetic manipulation, as Ortica *et al*. demonstrate^50^. Additionally, although studies of the liver transcriptome show *Dll1* is detectable but not highly expressed^61^, more sensitive methods may be required if expression is cell type-dependent, as our data suggest. Lastly, the observation of cholangiocytic differentiation localized at the periphery of cell islands in microarrays (Fig. 6B) is suggestive of currently undefined spatial localization mechanisms and underlying signaling gradients that could be systematically explored through future studies utilizing the microarray platform.

## Methods

### Cell culture, differentiation experiments, and treatments

BMEL 9A1 cells used in this study were between passages 26 and 35 and were cultured as previously described^37^. Briefly, cells were seeded on tissue culture plastic coated with collagen I (0.5 mg/ml) and cultured in an incubator under controlled conditions (37 °C and 5% CO_2_). Trypsin-EDTA (0.25% v/v) was used to detach cells for passaging. Basal (growth) media consisted of RPMI 1640 + GlutaMAX (Life Technologies, 61870-127) supplemented with fetal bovine serum (10% v/v, FBS), penicillin/streptomycin (1% v/v, P/S), and freshly-added human recombinant insulin (10 μg/ml, Life Technologies, 12585-014), IGF-2 (30 ng/ml, PeproTech, 100-12), and EGF (50 ng/ml, PeproTech, AF-100-15). Differentiation media consisted of Advanced RPMI 1640 (Life Technologies, 12633-012) supplemented with FBS (2% v/v), P/S (0.5% v/v), l-glutamine (1% v/v), and minimum non-essential amino acids (1% v/v, Life Technologies, 11140-050). During differentiation experiments, cells were seeded at 104E3 cells/cm^2^ and cultured for 72 h with a media change at 48 h unless otherwise noted. Differentiation experiments included the following treatments: TGFβ1 (1.5 ng/ml unless otherwise noted, R&D Systems, 240-B-002), GSI X (5 μM, EMD Millipore, 565771), and SB-431542 (10 μM, Sigma-Aldrich, S4317). For microarray experiments, cells were seeded at 2E6 cells/slide (106E3 cells/cm^2^) and allowed to adhere to patterned ECM domains for 2 h before washing 2× with media and adding experiment-specific treatments.

### shRNA lentivirus-mediated knockdown of Notch ligands

MISSION TRC shRNA lentiviral particles (Sigma-Aldrich) were used to transduce BMEL cells with a non-mammalian control sequence, *Jag1*-targeting sequence, and *Dll1*-targeting sequence per the manufacturer’s instructions at a multiplicity of infection (MOI) of 20–30 (see Supplemental Methods for TRC clone IDs and sequences). Cells were selected using puromycin (1.25 μg/ml) for 29–34 h after transduction (at which point untransduced cells were no longer viable) and subsequently cultured under reduced puromycin (0.625 μg/ml) for at least ~1–2 passages to avoid toxicity before banking in liquid nitrogen. qRT-PCR analysis indicated 67% knockdown for *Jag1* and 78% knockdown for *Dll1* while immunoblot further confirmed 73% knockdown for JAG1 (Supplemental Fig. S3). Puromycin selection was removed from the cells starting the passage before an experiment through endpoint.

### Immunoblot

Cell lysates were collected using ice-cold RIPA lysis buffer (Thermo Scientific, 89900) with an EDTA-free protease inhibitor cocktail (Thermo Scientific, 78425) per the manufacturer’s instructions. Samples were immediately pulse sonicated 3× and centrifuged at 14,000 × g for 15 min at 4 °C to remove cell debris. A BCA protein assay (Thermo Scientific, 23225) was performed in 96-well microplates per the manufacturer’s instructions to determine total protein concentrations. Isodiluted samples were further diluted in 4 × Laemmli sample buffer and 2-mercaptoethanol (50 mM), denatured at 95 °C for 5 min, and loaded into a pre-cast 4–20% polyacrylamide gel (Bio-Rad, 567–1093) at 50 μg/well. The gel was run in 1× tris/glycine/SDS at 200 V and 33–43 mA for 43–45 min. Transfer to a 0.45 μm PVDF membrane (EMD Millipore, IPVH00010) occurred in 1× tris/glycine and methanol (20% v/v) at 100 V using plate electrodes for 30 min, after which the membrane was placed in a blocking solution of non-fat dry milk (5% w/v) in wash buffer (1× tris-buffered saline and Tween-20 [0.05% w/v]) for 1 h with agitation. The membrane was subsequently incubated overnight on an orbital shaker at 4 °C in wash buffer with bovine serum albumin (5% w/v, BSA) and rabbit anti-JAG1 monoclonal antibody (56 ng/ml, 1/10,000 dilution from stock, Abcam, ab109536). After 3 × 10 min rinses with wash buffer, the membrane was incubated with a solution of HRP-linked anti-rabbit IgG (1/3,000 dilution from stock, Cell Signaling, 7074S) in wash buffer with non-fat dry milk (5% w/v) for 1 h at room temperature. The membrane was subsequently rinsed 6 × 5 min with wash buffer, incubated for 5 min with chemiluminescent substrate (Thermo Scientific, 34080), and imaged (ChemiDoc XRS Imaging System, Bio-Rad). To confirm equal protein loading, membranes were treated with stripping buffer (Thermo Scientific, 21059) and labeled with monoclonal rabbit anti-β-actin (1/1,000 from stock, Cell Signaling, 4970S) using the same protocol. Protein content was quantified with Quantity One software (Bio-Rad); background was automatically subtracted.

### RNA isolation and qRT-PCR analysis

Cell lysates were collected in TRIzol solution (Life Technologies, 15596-026) from which RNA was isolated using phenol-chloroform extraction per the manufacturer’s instructions. Samples were subsequently digested with DNAse (New England Biolabs, M0303S) at 37 °C for 30 min and cleaned using an RNeasy Mini Kit (Qiagen, 74104) per the manufacturer’s instructions. RNA concentration was obtained by UV spectroscopy using a NanoDrop ND-1000 (Thermo Scientific); samples with a 260 nm/280 nm absorbance ratio <1.8 were discarded. cDNA from isolated RNA (500 ng unless otherwise specified) was generated using the iScript cDNA synthesis kit (Bio-Rad, 170-8891) and mixed with SsoAdvanced Universal SYBR Green Supermix (Bio-Rad, 1725264) with pre-added primer pairs at a final concentration of 100 nM/primer, again per the manufacturer’s instructions. Primer pairs for each gene of interest were designed using the NCBI’s Primer-BLAST^62^ with a target T_m_ of 60 °C (see Supplemental Methods for GenBank accession numbers and sequences). Thermal cycling and measurement of amplification curves were performed on a CFX Connect Real-Time PCR Detection System (Bio-Rad). Expression (i.e., 2^−ΔΔCt^) analysis was performed in R using a custom script^63^. mRNA expression was calculated relative to *Hprt1* and control samples as indicated.

### Immunofluorescence

Before double immunofluorescence for ALB and OPN, cells were treated with brefeldin A (10 μg/ml, R&D Systems, 1231/5), an inhibitor of protein translocation to Golgi, for 2 h. Cells were then fixed in paraformaldehyde (4% v/v in 1× phosphate buffered saline [PBS]) for 15 min and permeabilized in Triton X-100 (0.25% v/v in 1× PBS). After 1 h at room temperature in blocking buffer (donkey serum [5% v/v] in 1× PBS), samples were incubated at room temperature with mouse anti-ALB (1/50 dilution from stock, R&D Systems, MAB1455) and goat anti-OPN (1/60 dilution from stock, R&D Systems, AF808) diluted in blocking buffer. After 3 × 5 min washes with 1× PBS, samples were incubated at room temperature with DyLight 550-conjugated donkey anti-mouse IgG (1/50 dilution from stock, Abcam, ab98767) and DyLight 488-conjugated donkey anti-goat IgG (1/50 dilution from stock, Abcam, ab96935). After another set of 3 × 5 min washes with 1× PBS, samples were mounted in Fluoromount G with DAPI (Southern Biotech, 0100-20). Immunofluorescence for arrayed proteins (namely JAG1 and DLL1) was performed as described above without the permeabilization and mounting steps; rabbit anti-JAG1 (1/50 dilution from stock, Abcam, ab109536), rabbit anti-DLL1 (1/200 dilution from stock, Santa Cruz Biotechnology, sc-9202), and donkey anti-rabbit IgG (1/200 dilution from stock, Abcam, ab96919) were used for these experiments. Samples were imaged with an Axiovert 200 M microscope (Carl Zeiss, Inc.) and associated Zen Pro software. The tiling feature of Zen Pro was used to compile images of entire microarrays.

### GFP+/GFP− co-cultures

Cells were infected with a CMV-driven hr-GFP adenovirus (University of Iowa Viral Vector Core Facility, Ad5CMVhr-GFP) at an MOI of 2,500 in differentiation media with polybrene (4 μg/ml) for 6 h, after which cells were cultured in growth media overnight. Both GFP+ and GFP− cells were passaged the next day and immediately co-cultured under differentiation conditions (TGFβ1±) at 96E3 GFP+ cells per 4.704E6 GFP− cells (a 1:50 ratio) in 3 × 100 mm petri dishes per combination of cell type (GFP+ or GFP−). Additional bulk monocultures of GFP− and GFP+ cell types were cultured in parallel to confirm initial basal state, differentiation capacity at mRNA transcript level, and expression of and sorting for GFP. After 72 h of culture, ≤100E3 GFP+ cells were collected using a FACSAria III sorter (BD Biosciences). RNA isolation and qRT-PCR analysis was then performed as described above with the amount of RNA varying between 150–300 ng depending on experimental yield.

### Microarray fabrication and characterization

Microarrays were fabricated as described previously^34^,^35^,^36^. Briefly, pre-cleaned microscope slides were silanized by treatment with 3-(trimethoxysilyl)propyl methacrylate (2% v/v) in ethanol for 30 min on an orbital shaker, after which slides were washed with ethanol for 5 min and baked on a hot plate at 110 °C. Our polyacrylamide pre-polymer solution consisted of acrylamide (10.55% w/v), bis-acrylamide (0.55% w/v), and Irgacure 2959 (2% w/v, BASF, 55047962) and was 0.2 μm-filtered and degassed as needed. Silanized slides were coated with 100 μl pre-polymer solution, covered with a 22 × 60 mm cover glass, and crosslinked using 365 nm UV A for 10 min (~240E3 μJ). Fabricated hydrogels were stored in excess dH_2_O with daily changes for three days and dehydrated on a hot plate at 50 °C for ~15 min. Biomolecules for arraying were diluted in 2× ECM protein buffer (38% v/v glycerol in dH_2_O, 16.4 mg/ml sodium acetate, 3.72 mg/ml EDTA, 0.5% v/v Triton X-100, ~80 ul glacial acetic acid, pH = 4.8) or 2× growth factor buffer (38% v/v glycerol in 1× PBS, 10.55 mg/ml sodium acetate, 3.72 mg/ml EDTA, 10 mg/ml CHAPS) and loaded in a 384-well V-bottom microplate. ECM proteins were prepared at a final total concentration of 250 μg/ml in 2× ECM protein buffer and included: collagen I (rat tail, EMD Millipore, 08–115), collagen III (human, EMD Millipore, CC054), collagen IV (human, EMD Millipore, CC076), fibronectin (human plasma, EMD Millipore, 341635), and laminin (mouse, EMD Millipore, CC095). Fc-recombinant Notch ligand solutions were prepared in 2× growth factor buffer and included: Fc-JAG1 (150 μg/ml final, R&D Systems, 599-JG-100), Fc-DLL1 (250 μg/ml final, R&D Systems, 5026-DL-050), and Fc-DLL4 (250 μg/ml final, Adipogen, AG-40A-0145-C050). All Notch ligand conditions were pre-conjugated with Protein A/G (Life Technologies, 21186) at a 1:6 molar ratio before arraying. Human IgG (970.6 μg/ml final, R&D Systems, 1-001-A) was arrayed as a control in experiments involving Notch ligands. A robotic benchtop microarrayer (OmniGrid Micro, Digilab) loaded with SMP3 Stealth microarray pins (ArrayIt) was used to transfer biomolecules from source plate to polyacrylamide hydrogel substrate, producing ~150 μm arrayed domains. Fabricated arrays were stored at room temperature and 65% RH overnight and sterilized the next morning with 30 min UVC while immersed in 1× PBS supplemented with 1% (v/v) P/S, after which cells were seeded on arrays as described above.

### Quantification and analysis of microarrays

Array images were pre-processed in ImageJ and Fiji, producing 8-bit TIFF files^64^,^65^. Image size was reduced to ≤100 MB by binning to reduce memory requirements during computational analysis. CellProfiler was used to identify all cells on the arrays and associated intensities in each channel for each cell^66^. Array locations were manually recorded for each image using dextran-rhodamine markers included in each array and used to automatically assign a grid location and arrayed condition for each identified cell. Each biological replicate included 2–3 technical replicates (i.e., individual arrays). Channel intensities and other single-cell measures were normalized using quantile normalization by biological replicate and propagated throughout the remaining analysis. R and the ggplot2 package were used to visualize results while the plyr package performed analytical calculations using a customized set of scripts^67^,^68^. The percentage of cells positive for ALB or OPN in each arrayed condition was calculated by defining a cutoff 2 s.d. above the mean of the treatment negative for that marker, i.e., TGFβ1− for OPN and TGFβ1+ for ALB. For the Notch ligand arrays, this comparison was performed against arrayed IgG.

### Statistical analyses

At least three biological replicates were performed for each experiment. Data are presented as mean ± s.e.m. Where noted, Student’s *t*-tests were performed in R comparing the groups of interest using options denoting a two-tailed, two-sample comparison with unequal variance. Multiple regression analyses were performed in R (see Supplemental Methods).

## Additional Information

**How to cite this article**: Kaylan, K. B. *et al*. Combinatorial microenvironmental regulation of liver progenitor differentiation by Notch ligands, TGFβ, and extracellular matrix. *Sci. Rep.* **6**, 23490; doi: 10.1038/srep23490 (2016).

## Supplementary Material

Supplementary Information
