# Supplementary material for: Combinatorial microenvironmental regulation of liver progenitor differentiation by Notch ligands, TGFβ, and extracellular matrix
Source: Sci Rep. 2016 Mar 30;6:23490. doi: 10.1038/srep23490 (PMC4812246; doi:10.1038/srep23490)
Supplement: Supplementary Information [file srep23490-s1.pdf]

Combinatorial microenvironmental regulation of liver progenitor differentiation by Notch ligands, TGFβ, and extracellular matrix

Authors

Kerim B. Kaylan,[1,2] Viktoriya Ermilova,[1,2] Ravi Chandra Yada,[1] Gregory H. Underhill[1,*]

Affiliations

[1]Department of Bioengineering, University of Illinois at Urbana-Champaign, Urbana, IL, 61801, USA

Contact Information

*Correspondence to Gregory H. Underhill, gunderhi@illinois.edu

Additional Title Page Footnotes

[2]Co-first author

Supplemental Figures

Supplemental Figure S1: Dose-response of Hnf4a and Sox9 to TGFβ1 treatment, related to Figure 1.

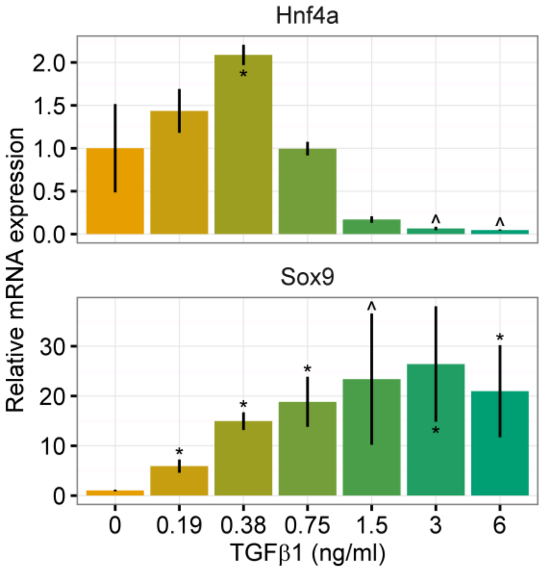

Data presented as mean ± s.e.m. with n=3. Student's *t*-tests were performed against 0 ng/ml for each concentration of TGFβ1 for *Hnf4a* and *Sox9* separately. P-values indicated for P<0.10 (^) and P<0.05 (*).

Supplemental Figure S2: Analysis of GSI X and SB-431542 treatments, related to Figures 1 and 2.

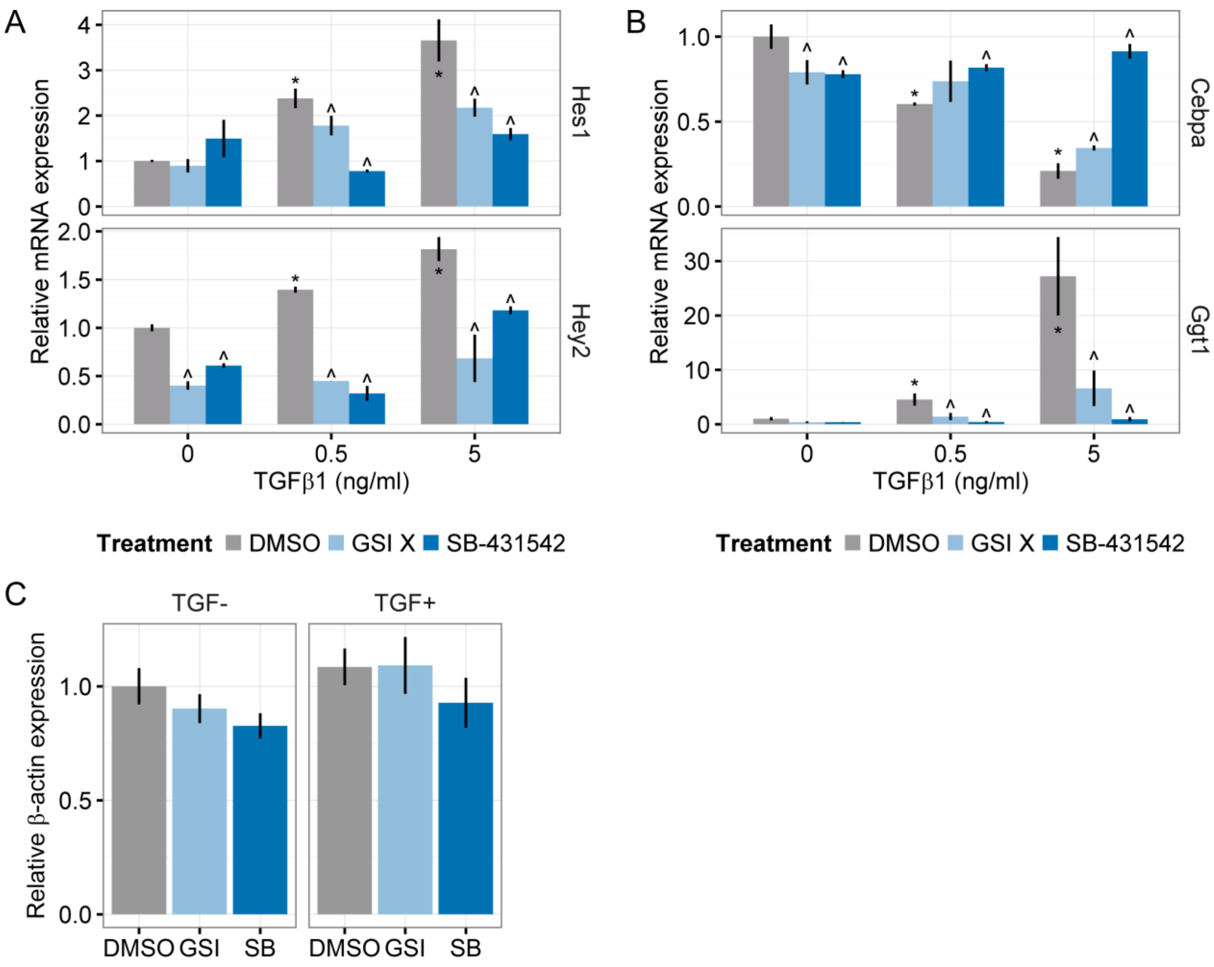

A. qRT-PCR analysis of mRNA transcripts of the Notch-related transcription factors *Hes1* and *Hey2* in BMEL cells treated with increasing doses of TGFβ1.

B. qRT-PCR analysis of mRNA transcripts of the hepatocytic transcription factor *Cebpa* and cholangiocytic marker *Ggt1* in BMEL cells treated with increasing doses of TGFβ1.

C. Quantification of β-actin protein expression (see Figures 2B and 2C) by immunoblot. n=3.

Data presented as mean ± s.e.m. with n≥2. In the DMSO treatment, Student's *t*-tests were performed against 0 ng/ml for each concentration of TGFβ1 for each gene separately with P-values indicated for $P<0.05$ (*). In the GSI X and SB-431542 treatments, Student's *t*-tests were performed against the corresponding DMSO treatment within each concentration of TGFβ1 with P-values indicated for $P<0.05$ (^).

Supplemental Figure S3: Expression and morphology of knockdown BMEL cells, related to Figure 2.

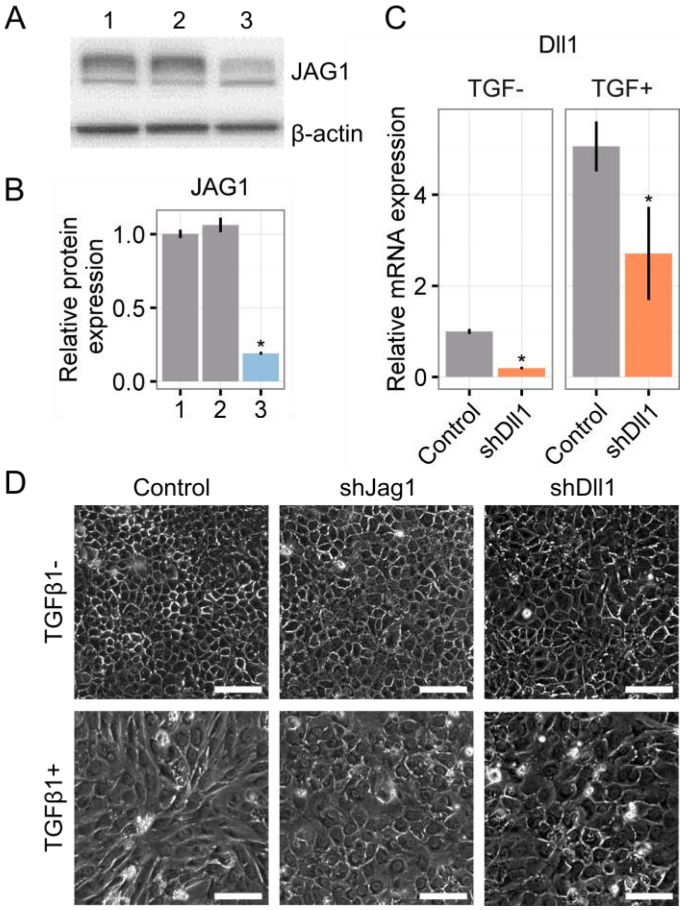

A. Representative immunoblot showing knockdown of JAG1 in shJag1 cells. 1: BMEL cells untransduced by lentiviral vector. 2: BMEL cells transduced with the control vector. 3: BMEL cells transduced with the shJag1 vector.
B. Quantification of the JAG1 immunoblots described in Supplemental Figure S3A. Student's *t*-tests were performed against untransduced cells for control and shJag1 cells.
C. qRT-PCR analysis of *Dll1* mRNA transcript levels in control- and shDll1-infected BMEL cells. Student's *t*-tests were performed against control cells in each differentiation condition (TGFβ1±).
D. Phase contrast micrographs of control-, shJag1-, and shDll1-infected BMEL cells. shJag1 and shDll1 cells exhibited differential morphology in TGFβ1+ indicating reduced capacity for transformation. Scale bars are 25 µm.

Data presented as mean ± s.e.m. with n=3. P-values indicated for $P<0.05$ (*).

Supplemental Figure S4: GFP+/GFP- co-cultures, related to Figure 3.

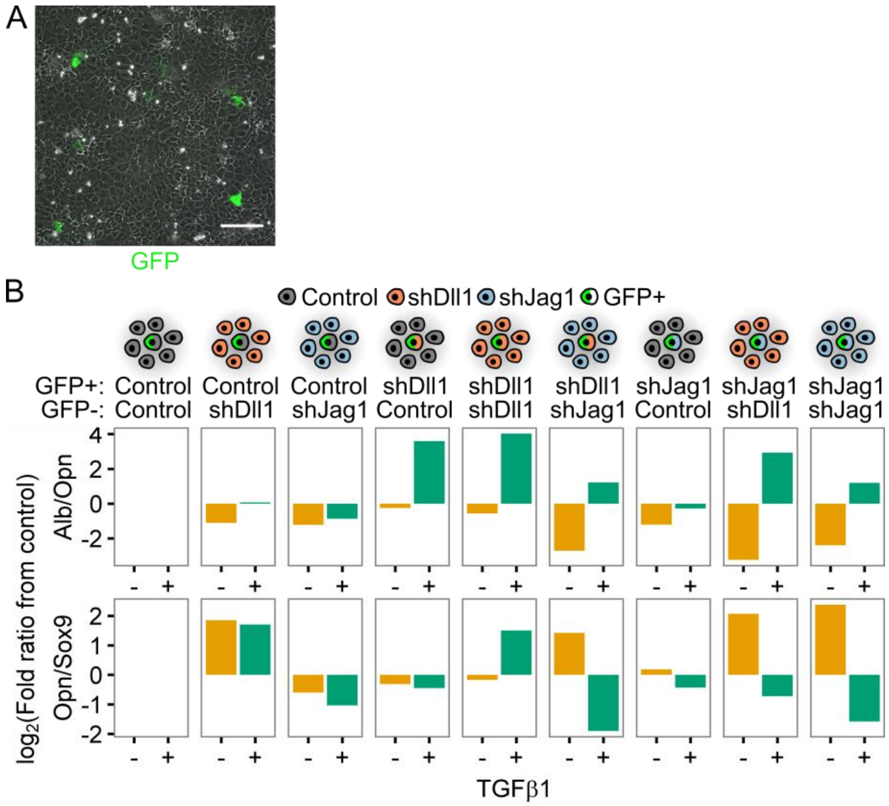

A. Combined immunofluorescence and phase contrast micrograph of GFP+/GFP- BMEL cells in TGFβ1- showing spatial separation of GFP+ cells. Scale bar is 200 μm.

B. Ratios of relative mRNA expression for *Alb*/*Opn* (hepatocytic/cholangiocytic) and *Opn*/*Sox9* normalized to control and log_2-transformed.

Data presented as mean. Error bars were not calculated for gene ratios.

Supplemental Figure S5: DAPI quantification of ECM arrays, related to Figure 5.

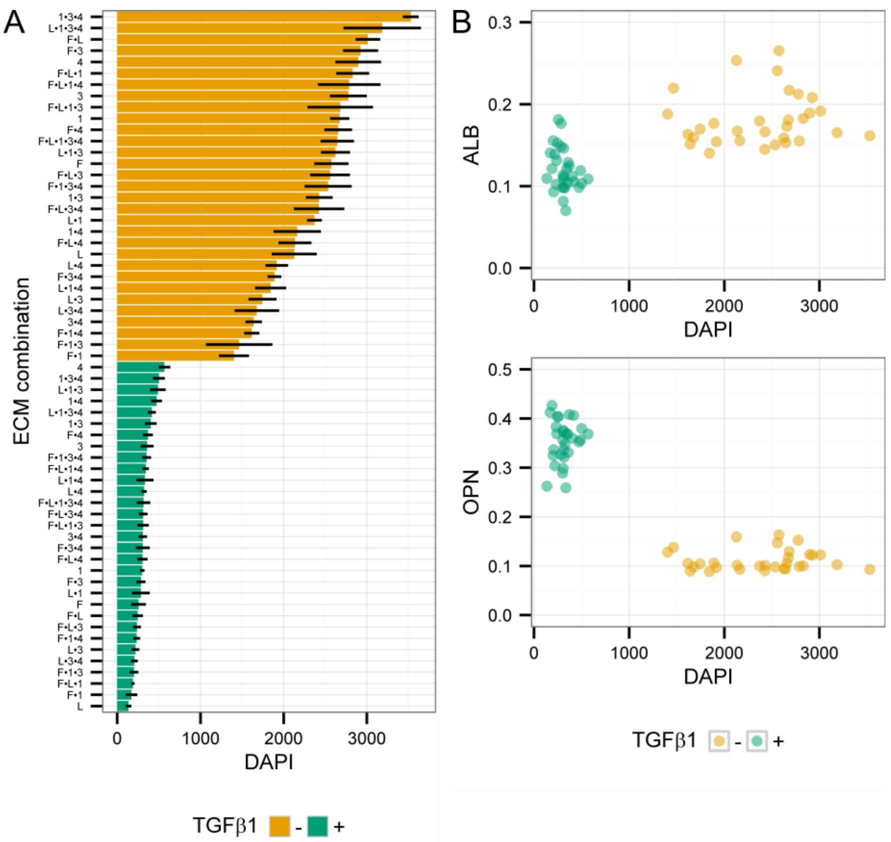

A. Sum of the intensity of the DAPI nuclear label (a proxy for cell count) on each ECM combination by treatment (TGFβ1±) showing decrease in TGFβ1+ compared to TGFβ1-.
B. Scatter plot of mean ALB intensity against DAPI by treatment (TGFβ1±). Each point represents a single arrayed ECM protein combination.
C. Scatter plot of mean OPN intensity against DAPI by treatment (TGFβ1±). Each point represents a single arrayed ECM protein combination.

Data presented as mean ± s.e.m. with n=3. Abbreviations: 1=collagen I, 3=collagen III, 4=collagen IV, F=fibronectin, L=laminin. Combinations denoted by "•", e.g., "1•3•4" denotes an ECM combination containing collagen I, III, and IV.

Supplemental Figure S6: ALB and OPN quantification of ECM arrays, related to Figure 5.

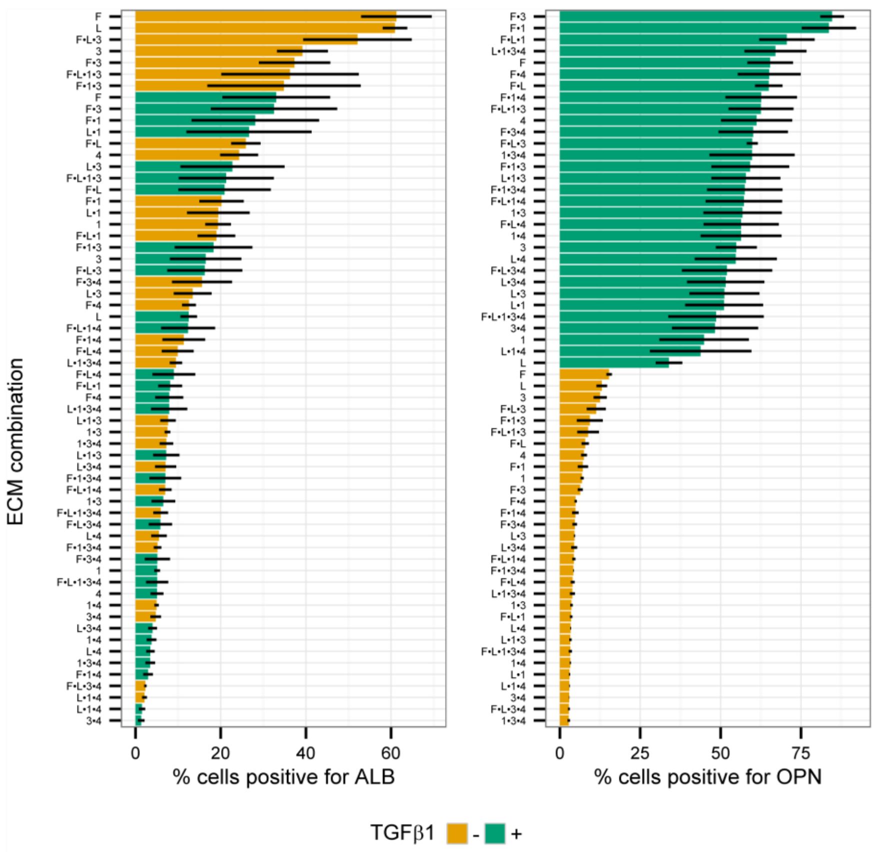

Quantification of microarrays of BMEL cells under differentiation conditions (TGFβ1±) on all 2^5 combinations of five ECM proteins. Data presented as mean ± s.e.m. with n=3. Abbreviations: 1=collagen I, 3=collagen III, 4=collagen IV, F=fibronectin, L=laminin. Combinations denoted by "•", e.g., "1•3•4" denotes an ECM combination containing collagen I, III, and IV.

Supplemental Figure S7: Effects from multiple regression analysis of ECM arrays, related to Figure 5.

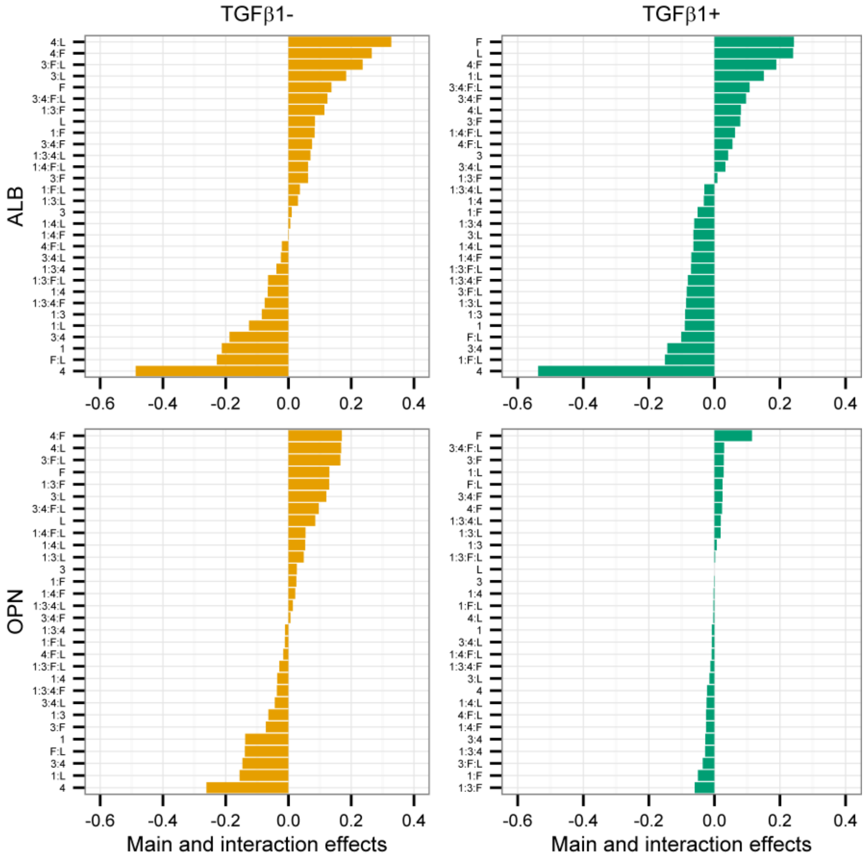

Main and interaction effects extracted from full factorial multiple regression analysis of each combination of immunolabeled protein (ALB and OPN by row) and treatment (TGFβ1± by column) in the ECM arrays. Abbreviations: 1=collagen I, 3=collagen III, 4=collagen IV, F=fibronectin, L=laminin. Interactions between factors are denoted by ":", e.g., "4:F" is the interaction effect of collagen IV and fibronectin. See Supplemental Methods for more information.

Supplemental Figure S8: Notch ligand arrays in TGFβ1+, related to Figure 6.

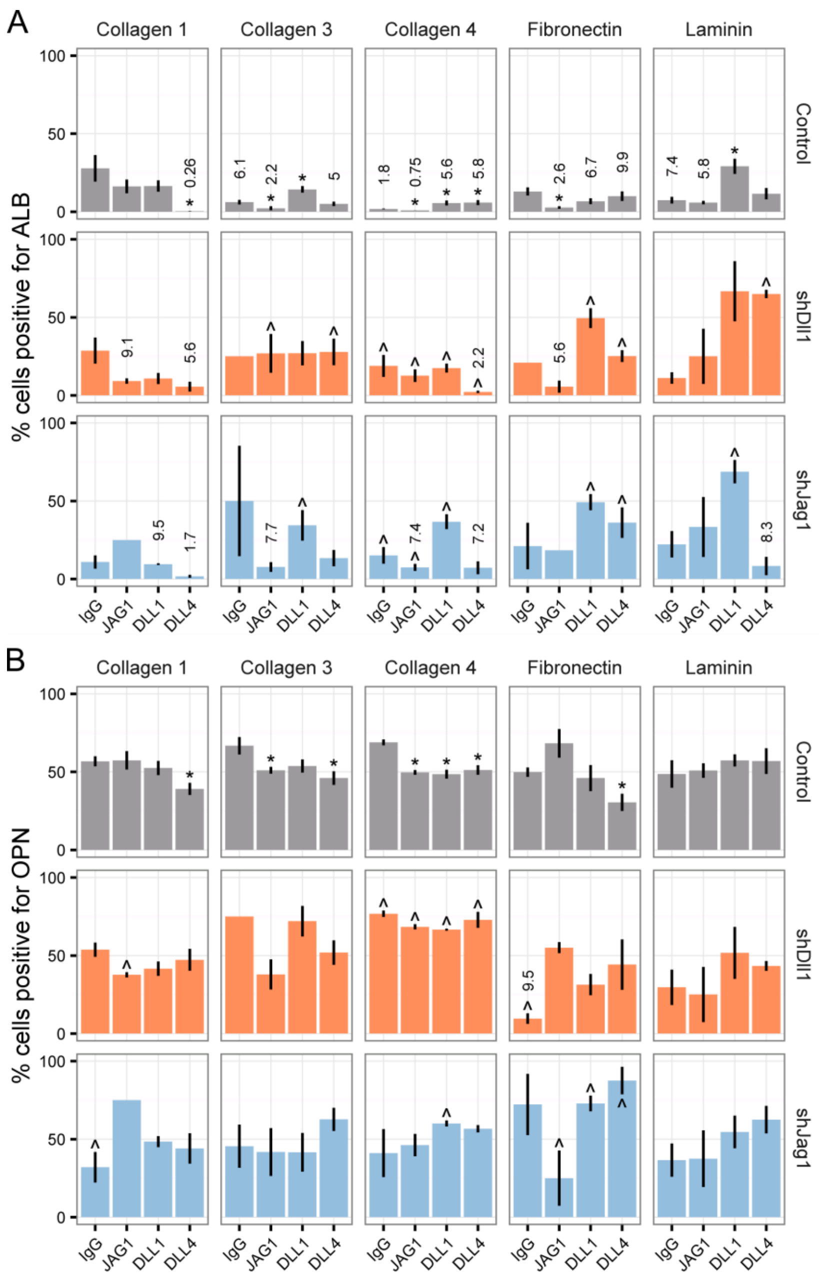

A. ALB quantification of shRNA-infected BMEL cells in TGFβ1+ on five ECM proteins.

B. OPN quantification of shRNA-infected BMEL cells in TGFβ1+ on five ECM proteins.

Data presented as mean ± s.e.m. with n=3. For control cells, Student's *t*-tests were performed against IgG for each arrayed Notch ligand within each ECM condition with P-values indicated for $P<0.05$ (*). For shDll1 and shJag1 cells, Student's *t*-tests were performed against the corresponding arrayed condition for control cells, again within each ECM condition with P-values indicated for $P<0.05$ (^). Numeric callouts show *y*-axis values (not P-values).

Supplemental Figure S9: Effects from regression analysis of Notch ligand arrays, related to Figure 6.

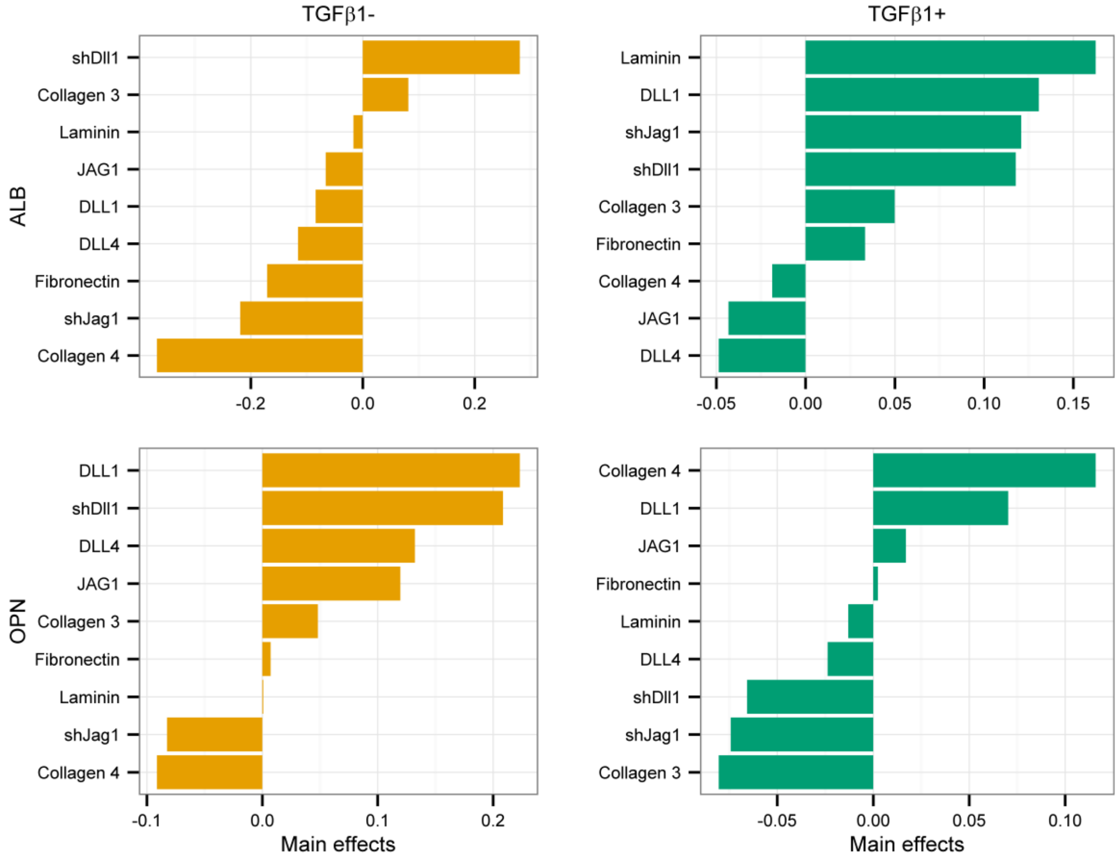

Main effects from multiple regression analysis of each combination of immunolabeled protein (ALB and OPN by row) and treatment condition (TGFβ1± by column) in the Notch ligand arrays. Collagen I, IgG, and non-target shRNA-infected (i.e., control) cells served as the reference factors. See Supplemental Methods for more information.

Supplemental Methods

Multiple regression analyses

Multiple regression analysis was performed in R using the base `lm` function. Both main and interaction effects were analyzed for the ECM arrays while only main effects were analyzed for the Notch ligand arrays. For all models, we confirmed homoscedasticity, normal distribution of residuals around zero, and absence of highly-influential outliers using residual-fit, Q-Q, and scale-location plots.

shRNA information

**Sigma Cat. No.**	**Description**	**Target or GenBank Accession**	**TRC Clone ID**	**Sequence (5'-to-3')**
SHC002H	MISSION pLKO.1-puro non-mammalian shRNA control lentiviral transduction particles	Non-mammalian sequences	N/A	CCGGCAACAAGATGAAGAGCACCAACTCGAGTTGGTGCTCTTCATCTTGTTGTTTTT
SHCLNV	MISSION pLKO.1-puro lentiviral transduction particles	NM_007865 (*Mus musculus Dll1*)	TRCN0000028865	CGCGAGAAGGACGTTTCTGTT
SHCLNV	MISSION pLKO.1-puro lentiviral transduction particles	NM_013822 (*Mus musculus Jag1*)	TRCN0000279260	GTGACGAAGCCACGTGTAATA

Primer pairs

Gene Symbol	GenBank Accession	Sequence (5'-to-3')
Alb	NM_009654.3	Forward: AAGCTGAGACCTTCACCTTCCACT<br>Reverse: CAGCTCAGCAAGAGCCGTTTGTTT
Cebpa	NM_007678	Forward: AGCCAAGAAGTCGGTGGACAAGAA<br>Reverse: TGGTCAACTCCAGCACCTTCTGTT
Dll1	NM_007865.3	Forward: ACGGAGAAGGTTGCTCTGTGTTCT<br>Reverse: CACTCCCCTGGTTTGTCACAGTAT
Dll4	NM_019454	Forward: TTCGTCGTCAGGGACAAGAATAGC<br>Reverse: CACAGCAAGAGAGCCTTGGATGAT
Ggt1	NM_008116	Forward: TGAGGTTATCAATGCCCGTGAGGT<br>Reverse: TAACCACGGATTTCACCAGGGACA
Hes1	NM_008235	Forward: CAACACGACACCGGACAAACCAAA<br>Reverse: TGGAATGCCGGGAGCTATCTTTCT
Hey2	NM_013904	Forward: CTTGTGAGGAAACGACCTCCGAAA<br>Reverse: ACCTCATCACTGAGCTTGTAGCGT
Hprt1	NM_013556.2	Forward: GGAGTCCTGTTGATGTTGCCAGTA<br>Reverse: GGGACGCAGCAACTGACATTTCTA
Jag1	NM_013822.5	Forward: CCGTAATCGCATCGTACTGCCTTT<br>Reverse: ATTGCCGGCTAGGGTTTATCATGC
Jag2	NM_010588	Forward: TGGGACAATGACACCACTCCAGAT<br>Reverse: AGTTCTCATCACAGCGTACTCGGA
Sox9	NM_011448.4	Forward: ACGGAACAGACTCACATCTCTCCT<br>Reverse: TCGCTTCAGATCAACTTTGCCAGC
Spp1 (*Opn*)	NM_009263.3	Forward: ACTACGACCATGAGATTGGCAGTG<br>Reverse: CTATAGGATCTGGGTGCAGGCTGTAAA
